# Pressure dependence of side chain ^1^H and ^15^N-chemical shifts in the model peptides Ac-Gly-Gly-Xxx-Ala-NH_2_

**DOI:** 10.1007/s10858-020-00326-w

**Published:** 2020-06-22

**Authors:** Markus Beck Erlach, Joerg Koehler, Claudia E. Munte, Werner Kremer, Edson Crusca, Masatsune Kainosho, Hans Robert Kalbitzer

**Affiliations:** 1 Institute of Biophysics and Physical Biochemistry and Centre of Magnetic Resonance in Chemistry and Biomedicine, University of Regensburg, 93040 Regensburg, Germany; 2 Physics Institute of São Carlos, University of São Paulo, São Carlos, 13566-590 Brazil; 3 Graduate School of Science, Tokyo Metropolitan University, 1-1 Minami-Ohsawa, Hachioji, Tokyo 192-0397 Japan

**Keywords:** High pressure NMR, Pressure coefficients, model peptides, Random-coil, Chemical shift, 1H, 15N, multi-state equilibria

## Abstract

For interpreting the pressure induced shifts of resonance lines of folded as well as unfolded proteins the availability of data from well-defined model systems is indispensable. Here, we report the pressure dependence of ^1^H and ^15^N chemical shifts of the side chain atoms in the protected tetrapeptides Ac-Gly-Gly-Xxx-Ala-NH_2_ (Xxx is one of the 20 canonical amino acids) measured at 800 MHz proton frequency. As observed earlier for other nuclei the chemical shifts of the side chain nuclei have a nonlinear dependence on pressure in the range from 0.1 to 200 MPa. The pressure response is described by a second degree polynomial with the pressure coefficients *B*_1_ and *B*_2_ that are dependent on the atom type and type of amino acid studied. A number of resonances could be assigned stereospecifically including the ^1^H and ^15^N resonances of the guanidine group of arginine. In addition, stereoselectively isotope labeled SAIL amino acids were used to support the stereochemical assignments. The random-coil pressure coefficients are also dependent on the neighbor in the sequence as an analysis of the data shows. For H^α^ and H^N^ correction factors for different amino acids were derived. In addition, a simple correction of compression effects in thermodynamic analysis of structural transitions in proteins was derived on the basis of random-coil pressure coefficients.

## Introduction

The study of the pressure response of polypeptides and proteins by high pressure NMR spectroscopy can be used for characterization of the free energy landscape of proteins (for reviews see e.g. Kitahara et al. 2013; Akasaka and Matsuki 2015). The pressure response allows the detection of rare “excited” conformational states of proteins that are important for folding and function (see e.g. Kalbitzer et al. 2009). Excited states also provide the basis for the development of a new type of allosteric inhibitors of proteins involved in signal transduction, called intrinsic allosteric inhibitors (Rosnizeck et al. 2010, 2012; Kalbitzer et al. 2013a; Kalbitzer and Spoerner b).

For the detection of rare states of proteins, mainly pressure dependent changes of chemical shifts are evaluated that are in many cases non-linear and can often be fitted with an appropriate thermodynamical model. These conformational contributions have to be separated from other chemical shift contributions as they are observed even in non-folded model compounds as a consequence of compression and rearrangement of the water shell. A non-linear pressure response of chemical shifts can even be determined experimentally in the peptide bond model *N*-methyl acetamide and is supported by quantum chemical calculations (Frach et al. 2016). However, random-coil peptides are better suited models for protein work. Arnold et al. (2002) reported the first data set for the main chain and side chain protons of the tetrapeptide Gly-Gly-Xxx-Ala in the pressure range up to 200 MPa. The pressure dependence of chemical shifts *δ* can sufficiently well be described by a second order polynomial with the chemical shift at pressure *P*_0_ (0.1 MPa) and the first and second order pressure coefficients *B*_1_ and *B*_2_. A data set recorded at 800 MHz was also published for all backbone atoms and the carbon resonances of the side chains of the protected tetrapeptide Ac-Gly-Gly-Xxx-Ala-NH_2_ (Koehler et al. 2012; Beck Erlach et al. 2016, 2017).

The quality of data by Arnold et al. (2002) measured at 600 MHz was not sufficient to determine the second order coefficient for the side chain protons. In this paper, we will present such data recorded at the N- and C-terminally protected tetrapeptide together with the pressure response of nitrogen side chain atoms not yet reported. With these data a complete data set for all nuclei of the model tetrapeptide Ac-Gly-Gly-Xxx-Ala-NH_2_ will be available for the scientific community.

## Materials and methods

### Synthesis of tetrapeptides

Uniformly ^13^C and ^15^N enriched and Fmoc (*N*-(9-Fluorenylmethoxycarbonyl)) protected amino acids required for the synthesis were purchased from Sigma Aldrich (St. Louis, MO, USA). The isotope enrichment is larger than 98%. All other chemicals were purchased from Merck (Darmstadt, Germany).

The synthesis of the tetrapeptide Ac-Gly-Gly-Xxx-Ala-NH_2_, where Xxx stands for one of the 20 canonical amino acids, was already described in detail earlier (Koehler et al. 2012; Beck Erlach et al. 2016). Only the amino acid Xxx at position 3 in the tetrapeptide Ac-Gly-Gly-Xxx-Ala-NH_2_ was uniformly ^13^C and ^15^N enriched. The purity of the tetrapeptides was confirmed by ESI–MS (Bruker, Billerica, MA, USA) and RP-HPLC (Waters, Milford, MA, USA).

### Selectively enriched SAIL amino acids

Stereoselectively ^2^H, ^15^N and ^13^C labeled amino acids were obtained from SAIL Technologies Inc. (Tokyo, Japan). For more details, see Kainosho et al. (2006), Kainosho and Güntert (2009).

### Sample preparation

Tetrapeptide samples were prepared by dissolving 2.5 µmol of the peptide in 500 µL of a buffer containing 20 mM perdeuterated Tris-d_11_ (Tris(hydroxymethyl-d_3_)amino-d_2_-methane) and 0.5 mM DSS (4,4-dimethyl-4-silapentane-1-sulfonic acid) with a ratio H_2_O:D_2_O of 90:10. Thus a final peptide concentration of 5 mM was obtained. The pH value was adjusted to 6.7 using a Hamilton Spintrode attached to a Beckman Coulter pH meter. Histidine was also measured at pH 4.0 and pH 8.5. The pH values have not been corrected for the deuterium isotope effect.

For the stereospecific assignment of amino acids, unlabeled amino acids or (stereo)-selectively ^2^H, ^13^C enriched SAIL amino acids were dissolved in 20 mM Tris-d_11_, 0.5 mM DSS and 10% D_2_O, pH 6.7, to obtain a final amino acid concentration of 4 to 10 mM.

### NMR spectroscopy

Most of the experiments were performed on an 800 MHz Bruker Avance spectrometer (Bruker, Billerica, MA, USA) with a room temperature probe head (QXI). The experiments were performed at 283 K, with a temperature calibration carried out after each sample change by measuring the difference of the proton resonance of the hydroxyl and the methyl group in 100% methanol as described by Raiford et al. (1979).

^1^H-NMR spectra were directly referenced to the methyl resonances of internal DSS, ^15^N signals were indirectly referenced to DSS using a Ξ-value of 0.101329118 (^15^N/^1^H) (Wishart et al. 1995b). Atom labels were named according to IUPAC recommendations (Markley et al. 1998).

^1^H and ^15^N chemical shifts were obtained from highly resolved 1D proton and 2D [^1^H, ^15^N]-HSQC spectra with a typical digital resolution of the time domain data of 0.04 Hz (^1^H) and 0.32 Hz (^15^N). A Lorentzian-to-Gaussian transformation was applied to the FID to obtain signals as narrow as possible.

Data acquisition and processing was performed with Bruker TopSpin 3.2 PL6. For peak picking the software AUREMOL (Gronwald and Kalbitzer, 2004) was used. Data evaluation and fitting was done with the software package R (R Core Team 2019).

### High pressure system

The high pressure system, especially the autoclave holding the ceramic cell was described in detail by Koehler et al. (2012). Pressure was applied to the NMR sample via pressurized fluids (methylcyclohexane or water) contained in high pressure lines. For generating the pressure a manually operated piston compressor and an air-to-liquid pressure intensifier (Barocycler®, HUB440, Pressure BioSciences Inc., South Easton, MA, USA), which is controlled by the spectrometer, were used. The ceramic cell was purchased from Daedalus Innovations LLC (Aston, PA, USA) with a maximum pressure limit of 250 MPa. For safety reasons pressure was only applied up to 200 MPa. The autoclave holding the ceramic cell is similar to the original autoclave (Peterson and Wand 2005) provided by Daedalus Innovations LLC but has an integrated safety valve, similar to the security valve described by Beck Erlach et al. (2010).

### Data evaluation

For all 20 model peptides a pressure series from 1 to 200 MPa was performed. The obtained pressure dependent chemical shifts *δ* were fitted as a function of pressure *P* with a second degree polynomial

1 $$ \begin{aligned}\delta \left(P\right)&=\delta \left({P}_{0}\right)+\frac{d\delta }{dP}\left({P}_{0}\right)\left(P-{P}_{0}\right)+\frac{1}{2}\frac{{d}^{2}\delta }{d{P}^{2}}\left({P}_{0}\right){\left(P-{P}_{0}\right)}^{2}\\&={\delta }_{0}+{B}_{1}\left(P-{P}_{0}\right)+{B}_{2}{\left(P-{P}_{0}\right)}^{2}\end{aligned} $$

with *P*_0_ the atmospheric pressure of 0.1 MPa and *δ*_0_ the chemical shift at pressure *P*_0_. *B*_1_ and *B*_2_ are the first and second order pressure coefficients. For random coil peptides, a second degree polynomial as defined by Eq. 1 is sufficient in the pressure range studied here (Arnold et al. 2002; Koehler et al. 2012; Beck Erlach et al. 2016, 2017). The description of the pressure response of small molecules such as the GTP analog GTPγS sometimes requires a third degree polynomial (Spoerner et al. 2017). When the ratio of *B*_2_/*B*_1_ is negative, an extremum with slope zero would be reached at a pressure *P*_ex_ with

2 $${P}_{ex}- {P}_{0}= -\frac{{B}_{1}}{{2 B}_{2}}$$

An extremum has not observed for our model peptides in the range up to 200 MP. With the mean value of *B*_2_/*B*_1_ for H^β^ (Table 2) of − 1.4 GPa^−1^ the maximum value would be expected at 375 MPa, far outside the pressure range studied. The dependence of the chemical shifts *δ* on pressure leads to an expression that is dependent on tanh (ΔG/2RT) (Beck Erlach et al. 2014). However, in a two state-model an extremum at high pressures is not expected from a thermodynamic description when ΔG is only linearly dependent on pressure *P*. This is the case when ΔG does not contain a second degree term, since he compressibility difference Δβ′ = -$\frac{\partial {\Delta V}_{ij}^{0}}{\partial P}=$ 0 as most authors assume in their data evaluation. The tanh function can be described by a second order differential equation typical for physical processes that show a saturation like behavior (Kepner 2010). In contrast to our second degree polynomial, it shows an asymptotic behavior at high pressures that we call saturation-like in the following. In fact, the Taylor-series of tanh itself has a second order term of zero, therefore the second degree polynomial that is traditionally used for a fit of the data is not suitable for the description of two-state equilibrium with ΔG only linearily dependent on *P*. However, when Δβ′ is not zero, again a maximum is obtained and a second degree term is required for a proper description of the data.

### Sequence dependent corrections for the pressure coefficients

Analogously to the method proposed by Schwarzinger et al. (2001) for the correction of the random-coil chemical shifts of atom *a* in amino acid *x* in position *i* by amino acid *y* in position *i* + *j* correction factors ${C}_{\mathrm{1,2}}^{-1,a}$(*y*), ${C}_{\mathrm{1,2}}^{+1,a}$(*y*), and ${C}_{\mathrm{1,2}}^{+2,a}$(*y*) for the pressure coefficients *B*_1_ and *B*_2_ were calculated. They can be obtained from the pressure response of Gly1, Gly2, and Ala4 by subtracting the *B*_1_ and *B*_2_ values obtained for Ac-GGGA-NH_2_ from those obtained for Ac-GGyA-NH_2_. The correction factors ${C}_{\mathrm{1,2}}^{-1,a}$(*y*) for the atom *a* (H^N^ or H^α^) of amino acid *x* in position *i* by amino acid *y* in position *i*-1 are given by

3 $${C}_{\mathrm{1,2}}^{-1,a}\left({y}\right)={B}_{\mathrm{1,2}}^{a}\left(\mathrm{A}\mathrm{c}-\mathrm{G}\mathrm{G}{y}\mathbf{A}-{\mathrm{N}\mathrm{H}}_{2}\right)-{B}_{\mathrm{1,2}}^{a}\left(\mathrm{A}\mathrm{c}-\mathrm{G}\mathrm{G}\mathrm{G}\mathbf{A}-{\mathrm{N}\mathrm{H}}_{2}\right)$$

with ${B}_{\mathrm{1,2}}^{a}$ the pressure coefficients of atom *a* in Ala4. Analogously, the correction factors ${C}_{\mathrm{1,2}}^{+1,a}$(*y*), and ${C}_{\mathrm{1,2}}^{+2,a}$(*y*) are obtained from Gly2 and Gly1, respectively. The sequence corrected *B*_1_ and *B*_2_ values ${B}_{\mathrm{1,2}}^{a,corr}$ for atom *a* in amino acid *x* in the sequence -*uxyz*- are than given as

4 $${B}_{\mathrm{1,2}}^{a,corr}\left({x}\right)={B}_{\mathrm{1,2}}^{a}\left({x}\right)+{C}_{\mathrm{1,2}}^{-1,a}\left({u}\right)+{C}_{\mathrm{1,2}}^{+1,a}\left({y}\right)+{C}_{\mathrm{1,2}}^{+2,a}\left({z}\right)$$

## Results and discussion

### Assignment of resonance lines

By applying pressure to the tetrapeptides and fitting the resulting pressure dependence of the chemical shift to Eq. 1, we obtained a complete dataset of ^1^H and ^15^N random coil chemical shift values for side chains of the amino acid 3 in the model peptides Ac-Gly-Gly-Xxx-Ala-NH_2_. The assignments of most ^1^H-resonances could be done on the basis of the already published proton assignments from Bundi and Wüthrich (1979) of Gly-Gly-Xxx-Ala and an analysis of the multiplet patterns. When necessary classical two-dimensional COSY, TOCSY and NOESY spectra were recorded. The ^15^N resonances could be assigned by HSQC-spectra from the adjacent protons already assigned.

### Stereospecific assignments

#### Geminal proton resonances in stereo selectively labeled SAIL amino acids

At 800 MHz proton resonance frequency most of the geminal proton in the tetrapeptides are resolved and can be observed separately. The typical geminal coupling constant in sp^3^ bonds is -12 Hz, corresponding to 0.015 ppm at 800 MHz. For most methylene protons the peak separation is significantly larger. They can be observed separately but still strong coupling effects are visible (see below). This is also true for the isolated amino acids (Table 1). Since stereo selectively deuterated SAIL amino acids (Kainosho et al. 2006; Kainosho and Güntert 2009) are available, at least in the amino acids the geminal proton resonances can unequivocally be assigned. When comparing the spectra of isotope labeled with the corresponding unlabeled amino acids, a difficulty is the isotope shift that occurs in the SAIL amino acids since these are not only stereoselectively deuterated but also ^15^N and ^13^C enriched. In general, these isotope shifts are smaller than the separation of the corresponding proton resonances. The isotope labelling induces an upfield shift of the order of 0.014 to 0.043 ppm (Table 1). Since deuteration is not 100% complete because of the limited purity of the starting materials, for many geminal protons a weak signal of the unlabeled group can be detected (values in brackets in Table 1). But even here an upfield isotope shift is observed caused by the other nuclei (especially also the ^13^C-nuclei of the methylene groups). When the resonances of geminal protons are separated, the H^β2^ resonances are always shifted upfield relative to the H^β3^ resonances. The only exception is cysteine. These relative shifts also apply for the methylene resonances in γ-position of Ile and Met. However, for the H^γ2^ and H^γ3^ resonances of Lys and the H^δ2^ and H^δ3^ resonances of Pro the order is interchanged. These assignments give also a hint to the stereospecific assignments in the protected tetrapeptides (Table 2) but cannot prove their stereospecific assignment definitively.

**Table 1:** Stereochemical assignments in SAIL amino acids^a^

Xxx	Atom	SAIL	Unlabeled	Difference
		*δ*_0_	*δ*_0_	Δ*δ*_0_^b^
		(ppm)	(ppm)	(ppm)
Arg	H^β2^	(1.862)	1.898	0.036
	H^β3^	1.881	1.898	0.017
	H^γ2^	(−^c^)	1.708	(−^c^)
	H^γ3^	1.594	1.629	0.035
	H^δ2/δ3^	3.205	3.233	0.028
Asn	H^β2^	2.820	2.842	0.022
	H^β3^	(2.918)	2.939	0.021
Asp	H^β2^	2.641	2.661	0.020
	H^β3^	(−^c^)	2.805	(−^c^)
Cys_red_	H^β2^	3.070	3.090	0.020
	H^β3^	(−^c^)	3.010	(−^c^)
Cys_ox_	H^β2^	3.137	3.160	0.023
	H^β3^	(−^c^)	3.374	(−^c^)
Gln	H^β2/β3^	2.095	2.122	0.027
	H^γ2/γ3^	2.422	2.437	0.015
Glu	H^β2^	2.020	2.050	0.030
	H^β3^	(2.103)	2.118	0.015
	H^γ2/γ3^	2.323	2.339	0.016
His	H^β2^	3.103	3.129	0.026
	H^β3^	(−^c^)	3.226	(−^c^)
Ile	H^γ12^	1.206	1.247	0.041
	H^γ13^	(1.414)	1.451	0.037
Leu	H^β2^	(−^c^)	1.677	(−^c^)
	H^β3^	1.693	1.713	0.020
	H^γ^	1.652	1.691	0.039
Lys	H^β2/β3^	1.864	1.889	0.025
	H^γ2^	(1.459)	1.489	0.030
	H^γ3^	1.376	1.419	0.043
	H^δ2/δ3^	1.674	1.709	0.035
	H^ε2 ε3^	2.984	3.009	0.025
Met	H^β2^	2.085	2.117	0.032
	H^β3^	(2.160)	2.182	0.022
	H^γ2/γ3^	2.592	2.623	0.031
Phe	H^β2^	(−^c^)	3.110	(−^c^)
	H^β3^	3.255	3.272	0.017
Pro	H^β2^	2.028	2.054	0.026
	H^β3^	(2.325)	2.339	0.014
	H^γ2/γ3^	1.950	1.996	0.046
	H^δ2^	3.386	3.406	0.020
	H^δ3^	(3.301)	3.320	0.019
Ser	H^β2^	(3.916)	3.935	0.019
	H^β3^	3.948	3.971	0.023
Trp	H^β2^	(−^c^)	3.293	(−^c^)
	H^β3^	3.454	3.473	0.019
Tyr	H^β2^	(3.026)	3.040	0.014
	H^β3^	3.166	3.185	0.019

^a^Values in brackets, remaining ^1^H signals from incomplete deuteration. Temperature 283 K, 800 MHz proton frequency, 4 to 10 mM amino acid in 20 mM Tris-d_11_ pH 6.7, 0.5 mM DSS, 10% D_2_O

^b^Δ*δ*_0_ = *δ*_0_(unlabeled)–*δ*_0_(SAIL)

^c^Not detected

**Table 2:** Pressure dependence of the H^β^-chemical shifts^a^

Xxx	Atom	*δ*_0_	*B*_1_	*B*_2_	*B*_*2*_*/B*_*1*_
		(ppm)	(ppm GPa^−1^)	(ppm GPa^−2^)	(GPa^−1^)
Ala	(H^β^)_3_	1.392	− 0.022 ± 0.001	0.02 ± 0.01	− 0.91 ± 0.46
Arg	H^β2/β3^	1.769	− 0.026 ± 0.005	0.00 ± 0.02	0.00 ± −
	H^β2/β3^	1.874	− 0.077 ± 0.005	0.03 ± 0.02	− 0.39 ± 0.26
Asn	H^β2 b^	2.839	0.042 ± 0.011	− 0.13 ± 0.06	− 3.10 ± 1.64
	H^β3 b^	2.746	0.072 ± 0.014	− 0.12 ± 0.07	− 1.67 ± 1.03
Asp	H^β2 c^	2.623	0.083 ± 0.008	− 0.06 ± 0.04	− 0.72 ± 0.49
	H^β3 c^	2.704	0.073 ± 0.007	− 0.05 ± 0.03	− 0.68 ± 0.42
Cys_red_	H^β2^	2.938	− 0.001 ± 0.001	− 0.01 ± 0.01	(10.0 ± 14.1)
	H^β3^	2.938	− 0.001 ± 0.001	− 0.01 ± 0.01	(10.0 ± 14.1)
Cys_ox_	H^β2/β3^	2.982	0.053 ± 0.007	− 0.03 ± 0.03	(− 0.49 ± 0.55)
	H^β2/β3^	3.256	− 0.050 ± 0.001	0.03 ± 0.01	− 0.52 ± 0.14
Gln	H^β2 b^	2.133	− 0.099 ± 0.013	0.09 ± 0.06	− 0.91 ± 0.62
	H^β3 b^	1.987	− 0.041 ± 0.010	0.05 ± 0.05	(− 1.22 ± 1.26)
Glu	H^β2 c^	1.948	0.018 ± 0.010	− 0.11 ± 0.05	− 6.11 ± 4.39
	H^β3 c^	2.095	− 0.190 ± 0.035	0.24 ± 0.17	− 1.26 ± 0.92
His	H^β2 c^	3.163	0.020 ± 0.003	− 0.01 ± 0.01	(− 0.5 ± 0.5)
(pH 4.0)	H^β3 c^	3.284	− 0.085 ± 0.010	0.18 ± 0.05	− 2.12 ± 0.64
His	H^β2 c^	3.036	0.027 ± 0.012	− 0.02 ± 0.06	(− 0.74 ± 2.25)
(pH 8.5)	H^β3 c^	3.101	− 0.059 ± 0.008	0.10 ± 0.04	− 1.69 ± 0.72
Ile	H^β^	1.881	− 0.018 ± 0.006	0.04 ± 0.03	− 2.22 ± 1.82
Leu	H^β2 c^	1.584	− 0.028 ± 0.001	0.04 ± 0.01	− 1.43 ± 0.36
	H^β3 c^	1.652	− 0.048 ± 0.001	0.04 ± 0.01	− 0.83 ± 0.21
Lys	H^β2/β3^	1.758	− 0.041 ± 0.002	0.02 ± 0.01	− 0.49 ± 0.29
	H^β2/β3^	1.846	− 0.103 ± 0.007	0.07 ± 0.03	− 0.68 ± 0.39
Met	H^β2 c^	2.004	− 0.026 ± 0.003	0.02 ± 0.01	− 0.77 ± 0.40
	H^β3 c^	2.114	− 0.087 ± 0.005	0.10 ± 0.02	− 1.15 ± 0.24
Phe	H^β2 c^	3.049	0.013 ± 0.010	− 0.02 ± 0.05	(− 1.54 ± 4.02)
	H^β3 c^	3.129	− 0.049 ± 0.007	0.14 ± 0.03	− 2.86 ± 0.74
Pro_cis_	H^β2 c^	2.189	− 0.083 ± 0.013	0.10 ± 0.06	− 1.20 ± 0.75
	bH^β3 c^	2.359	− 0.076 ± 0.009	0.08 ± 0.04	− 1.05 ± 0.54
Pro_trans_	H^β2 c^	2.033	− 0.078 ± 0.009	0.13 ± 0.04	− 1.67 ± 0.55
	H^β3 c^	2.205	− 0.070 ± 0.010	0.10 ± 0.05	− 1.43 ± 0.74
Ser	H^β2/β3^	3.881	− 0.015 ± 0.005	− 0.01 ± 0.03	(0.67 ± 2.01)
	H^β2/β3^	3.881	− 0.015 ± 0.005	0.03 ± 0.03	(− 2.00 ± 2.11)
Thr	H^β^	4.255	0.001 ± 0.003	0.06 ± 0.01	(60 ± 180)
Trp	H^β2^	3.277	− 0.024 ± 0.008	0.02 ± 0.04	(− 0.83 ± 1.69)
	H^β3^	3.277	− 0.024 ± 0.008	0.02 ± 0.04	(− 0.83 ± 1.69)
Tyr	H^β2 c^	2.972	0.037 ± 0.010	− 0.13 ± 0.05	− 3.51 ± 1.65
	H^β3 c^	3.037	− 0.090 ± 0.019	0.34 ± 0.09	− 3.78 ± 1.28
Val	H^β^	2.093	0.007 ± 0.004	− 0.09 ± 0.02	− 12.86 ± 7.88
Mean^d^		2.58 (0.70)	− 0.027 (0.056)	0.03 (0.09)	0.4 (10.2)
					− 1.38 ± 0.16^e^

^a^*δ*^0^, *B*_1_, and *B*_2_ were obtained by a fit of the data to Eq. 1. The sample contained 5 mM tetrapeptide in 20 mM perdeuterated Tris-d_11_, 0.5 mM DSS, H_2_O/D_2_O 9:1. Temperature 283 K. The pH value was adjusted to 6.7. The errors correspond to a confidence level of 95%. A *B*_2_/*B*_1_-value is given in brackets when its error is equal/larger than the value itself

^b^Stereospecific assignments from Harsch et al. (2013)

^c^Tentative stereospecific assignment taken from free amino acids (Table 1)

^d^Mean values with standard deviation in brackets

^e^*B*_2_/*B*_1_ determined from the slope in the correlation plot (Fig. 4)

#### Stereochemical assignment of H^β^-proton resonances in Ac-GGXA-NH_2_

Also in the protected tetrapeptides, a large number of geminal proton resonances are non-equivalent at 800 MHz proton resonance frequency and can be observed separately (Table 2). However, in literature most of them were not assigned stereospecifically, since the high internal mobility in random coil peptides does complicate the use of simple NMR methods developed for folded proteins that are based on the assumption of a single conformer in solution. Here, only a combination of 2D-NOESY-spectroscopy with extended molecular dynamic runs allows a safe assignment, as previously reported by Harsch et al. (2013) for GGNA-NH_2_ and GGQA-NH_2_. Although our tetrapeptides are additionally acetylated at N-terminus, these assignments can safely be transferred to our peptides since the N-terminal protection has only minor effects on the chemical shifts of the β-methylene groups (GGNA-NH_2_: 2.84 ppm, 2.75 ppm; Ac-GGNA-NH_2_: 2.84 ppm, 2.75 ppm; GGQA-NH_2_: 2.12 ppm, 1.99 ppm; Ac-GGQA-NH_2_; 2.13 ppm, 1.99 ppm). However, the SAIL data do not further corroborate these assignments. The H^β2^ resonances of glutamine are not separated in the free amino acid glutamine. The H^β2^ resonance in Ac-GGNA-NH_2_ has the same chemical shift as in the isolated amino acid (2.84 ppm) but instead of being upfield shifted the H^β3^ resonance is strongly downfield shifted by 0.19 ppm (Tables 1 and 2), indicating that other factors influencing the chemical shifts play a role here. A comparison of H^β^ chemical shifts in the tetrapeptide and the SAIL amino acids allows a tentative stereochemical assignment of the resonances in Asp, Glu, His, Leu, Met, Phe, Pro, and Tyr. However, one has to be careful when using this assignment method since the peak separation is not very large: In Asn, one would have ended up with the wrong decision by just using the order of the chemical shifts (see above) in the free amino acid.

#### Methyl groups of Val and Leu

The stereochemical assignments of the methyl carbon resonances of Leu and Val in our tetrapeptides by selective deuteration were reported earlier by Beck Erlach et al. (2017). The assignment of corresponding proton resonances can be made directly from these data (see Table 3).

**Table 3:** Pressure dependence of chemical shifts of other carbon bound side chain proton resonances^a^

Xxx	Atom	*δ*_0_	*B*_1_	*B*_2_	*B*_*2*_*/B*_*1*_
		(ppm)	(ppm GPa^−1^)	(ppm GPa^−2^)	(GPa^−1^)
Arg	H^γ2^	1.633	− 0.104 ± 0.009	0.06 ± 0.04	− 0.58 ± 0.39
	H^γ3^	1.633	− 0.104 ± 0.009	0.06 ± 0.04	− 0.58 ± 0.39
	H^δ2^	3.204	− 0.088 ± 0.007	0.04 ± 0.03	− 0.45 ± 0.34
	H^δ3^	3.204	− 0.088 ± 0.007	0.04 ± 0.03	− 0.45 ± 0.34
Gln	H^γ2^	2.371	− 0.037 ± 0.010	− 0.01 ± 0.05	(0.27 ± 1.35)
	H^γ3^	2.371	− 0.037 ± 0.010	− 0.01 ± 0.05	(0.27 ± 1.35)
Glu	H^γ2^	2.324	− 0.078 ± 0.014	− 0.11 ± 0.07	1.41 ± 0.93
	H ^γ3^	2.324	− 0.078 ± 0.014	− 0.11 ± 0.07	1.41 ± 0.93
His	H^δ2^	7.284	0.013 ± 0.005	0.01 ± 0.02	(0.77 ± 1.57)
(pH 4.0)	H^ε1^	8.598	0.064 ± 0.003	− 0.03 ± 0.01	− 0.47 ± 0.16
His	H^δ2^	6.971	0.094 ± 0.057	− 0.23 ± 0.28	(− 2.45 ± 3.33)
(pH 8.5)	H^ε1^	7.702	0.137 ± 0.061	0.07 ± 0.30	0.51 ± 2.20
Ile	H^γ12 b^	1.188	− 0.117 ± 0.020	0.18 ± 0.10	− 1.54 ± 0.89
	H^γ13 b^	1.452	− 0.143 ± 0.018	− 0.12 ± 0.09	0.84 ± 0.64
	(H^γ2^)_3_	0.924	− 0.087 ± 0.005	0.04 ± 0.03	− 0.46 ± 0.35
	(H^δ1^)_3_	0.866	− 0.099 ± 0.006	0.07 ± 0.03	− 0.71 ± 0.31
Leu	H^γ^	1.634	− 0.125 ± 0.002	− 0.005 ± 0.008	(0.04 ± 0.06)
	(H^δ1^)_3_^c^	0.928	− 0.070 ± 0.001	0.030 ± 0.005	− 0.43 ± 0.07
	(H^δ2^)_3_^c^	0.874	− 0.064 ± 0.002	0.01 ± 0.01	(− 0.16 ± 0.16)
Lys	H^γ2^	1.433	− 0.159 ± 0.012	0.15 ± 0.06	− 0.94 ± 0.38
	H^γ3^	1.433	− 0.159 ± 0.012	0.15 ± 0.06	− 0.94 ± 0.38
	H^δ2^	1.678	− 0.108 ± 0.007	0.06 ± 0.03	− 0.56 ± 0.28
	H^δ3^	1.678	− 0.108 ± 0.007	0.06 ± 0.03	− 0.56 ± 0.28
	H^ε2^	2.989	− 0.071 ± 0.003	0.03 ± 0.01	− 0.42 ± 0.14
	H^ε3^	2.989	− 0.071 ± 0.003	0.03 ± 0.01	− 0.42 ± 0.14
Met	H^γ2/γ3^	2.542	− 0.109 ± 0.004	0.05 ± 0.02	− 0.46 ± 0.18
	H^γ2/γ3^	2.614	− 0.131 ± 0.002	0.13 ± 0.01	− 0.99 ± 0.08
	(H^ε^)_3_	2.098	− 0.070 ± 0.003	0.04 ± 0.01	− 0.57 ± 0.14
Phe	H^δ1/δ2^	7.277	− 0.065 ± 0.007	0.15 ± 0.03	− 2.31 ± 0.52
	H^ε1/ε2^	7.375	− 0.019 ± 0.008	0.12 ± 0.04	− 6.32 ± 3.39
	H^ζ^	7.346	− 0.008 ± 0.009	0.10 ± 0.04	(− 12.5 ± 14.9)
Pro_cis_	H^γ2/γ3^	1.848	− 0.162 ± 0.010	0.24 ± 0.05	− 1.48 ± 0.32
	H^γ2/γ3^	1.943	− 0.076 ± 0.010	0.15 ± 0.05	− 1.97 ± 0.71
	H^δ2/ δ3^	3.530	− 0.121 ± 0.005	0.12 ± 0.02	− 0.99 ± 0.17
	H^δ2/δ3^	3.575	− 0.079 ± 0.005	0.11 ± 0.02	− 1.39 ± 0.27
Pro_trans_	H^γ2^	2.021	− 0.110 ± 0.005	0.12 ± 0.02	− 1.09 ± 0.19
	H^γ3^	2.021	− 0.110 ± 0.005	0.12 ± 0.02	− 1.09 ± 0.19
	H^δ2^	3.638	− 0.089 ± 0.020	− 0.05 ± 0.10	(0.56 ± 1.13)
	H^δ3^	3.638	− 0.089 ± 0.020	− 0.05 ± 0.10	(0.56 ± 1.13)
Thr	(H^γ^)_3_	1.212	− 0.049 ± 0.002	0.02 ± 0.01	− 0.41 ± 0.20
Trp	H^δ1^	7.264	0.015 ± 0.009	0.19 ± 0.05	12.67 ± 8.30
	H^ε3^	7.642	− 0.113 ± 0.010	0.08 ± 0.05	− 0.71 ± 0.45
	H^ζ2^	7.498	0.003 ± 0.006	0.08 ± 0.03	(26.7 ± 54.3)
	H^ζ3^	7.171	− 0.047 ± 0.014	0.12 ± 0.07	− 2.55 ± 1.67
	H^η2^	7.247	− 0.030 ± 0.018	0.14 ± 0.09	− 4.67 ± 4.10
Tyr	H^δ1/δ2^	7.139	− 0.058 ± 0.007	0.09 ± 0.03	− 1.55 ± .55
	H^ε1/ε2^	6.848	− 0.023 ± 0.005	0.10 ± 0.03	− 4.35 ± 1.61
Val	(H^γ1^)_3_^c^	0.945	− 0.067 ± 0.002	0.04 ± 0.01	− 0.60 ± 0.15
	(H^γ2^)_3_^c^	0.931	− 0.068 ± 0.003	0.04 ± 0.01	− 0.59 ± 0.15
Mean^d^		3.57 (2.55)	− 0.069 (0.061)	0.06 (0.09)	− 0.3 (4.9)

^a^*δ*^0^, *B*_1_, and *B*_2_ were obtained by a fit of the data to Eq. 1. The errors correspond to a confidence level of 95%. Experimental conditions see Table 2. A *B*_2_/*B*_1_ –value is given in brackets when its error is equal/larger than the value itself

^b^Tentative stereospecific assignment taken from free amino acids (Table 1)

^c^Stereospecific assignments from Beck Erlach et al. (2017) using stereoselectively isotope labelled tetrapeptides

^d^Mean values with standard deviation in brackets

#### Amide and amino groups of Asn, Gln, and Arg

The stereochemical assignments of the side chain and C-terminal amide groups of Asn and Gln were earlier reported for the C-terminal protected tetrapeptides GGNA-NH_2_ and GGQA-NH_2_ by Harsch et al. (2013). As already stated above, our tetrapeptides are additionally acetylated at N-terminus. These assignments can safely transferred since the N-terminal protection has only minor effects on the chemical shifts. For the amide groups of GGNA-NH_2_ and Ac-GGNA-NH_2_ the shifts are (7.65, 6.96) ppm and (7.69, 6.99) ppm, respectively. The corresponding values of GGQA-NH_2_ and Ac-GGQA-NH_2_ are (7.59, 6.90) ppm and (7.64, 6.94) ppm, respectively. In fact, a general analysis of the BMRB data base shows that also in folded proteins the downfield shifted resonance lines can be assigned to H^δ21^ and H^ε21^, with a separation of the chemical shifts of the two amide resonance lines s ≥ 0.40 ppm for asparagine and ≥ 0.42 ppm for glutamine, at a confidence level > 95% (Harsch et al. 2017). In the past, the proton and nitrogen resonances of the guanidino group of Arg in the tetrapeptides have not been stereospecifically assigned, since at ambient temperature the moderately fast flip around the N–C-bonds averages expected NOEs between the H^ε^-proton and the H^η21^-protons. In the amino acid Arg the flip rate around the N^ε^–C^ζ^-bond is about 900 to 1000 s^−1^ at room temperature in the presence of 30% methanol-d_6_ (Henry and Sykes, 1995). As a consequence, the chemical shifts of the H^η^-protons are averaged at 500 MHz at room temperature. At 263 K and at 500 MHz proton resonance frequency the rotation around the N^ε^–C^ζ^-bond is sufficiently slowed down in this solution for observing two separated H^η^/N^η^-cross peaks at (6.52, 70.78) ppm and at (6.97, 72.78) ppm. At 223 K in the presence of 50% methanol the rotation rate around the C^ζ^-N^η^-bond is decreased in such a way that the two H^η^ signals bound to the downfield shifted nitrogen can be observed separately but not those of the highfield shifted nitrogen (Yamazaki et al. 1995). Unfortunately, stereospecific assignments have not been reported. In our tetrapeptide, the guanidino proton and nitrogen resonances are well separated at 283 K and at 800 MHz ^1^H-resonance frequency. A stereospecific NOE-based assignment cannot be performed at this temperature because of the motional averaging of the NOEs. However, the motions can be slowed down sufficiently by decreasing the pH to 2.4 and by decreasing the temperature to 260 K at 195 MPa where the solvent is still fluid. In the 3D-[^1^H, ^15^N]-NOESY-HSQC it shows a strong NOE from the H^ε^ resonance at 7.29 ppm to one set of the H^η^-resonances (data not shown). This indicates that the downfield shifted resonance at 6.98 ppm corresponds to H^η21/η22^ bound to N^η2^ under these experimental conditions. The assignment of the resonances at ambient conditions (Table 4) was performed by following the continuous temperature and pressure dependent shift changes. Interestingly, the H^η21/η22^ resonance corresponds to the downfield shifted resonance that shows a smaller rotation rate around the C^ζ^–N^η^-bond than the high field shifted resonance. This is in line with a small sterical hindrance by the N^ε^-group.

**Table 4:** Pressure induced shifts in side chain nitrogen groups and their directly bonded protons^a^

Xxx	Atom	*δ*_0_	*B*_1_	*B*_2_	*B*_*2*_*/B*_*1*_
		(ppm)	(ppm GPa^−1^)	(ppm GPa^−2^)	(GPa^−1^)
Arg	N^ε^	84.34	1.8 ± 0.3	− 4 ± 2	− 2.22 ± 1.17
	H^ε^	7.245	− 0.14 ± 0.01	− 0.13 ± 0.07	0.93 ± 0.50
	N^η1^	70.48	5.4 ± 0.2	− 6.0 ± 0.9	− 1.11 ± 0.17
	H^η11/η12^	6.490	0.025 ± 0.001	− 0.02 ± 0.01	− 0.80 ± 0.40
	N^η2^	71.78	5.6 ± 0.2	− 8.8 ± 0.9	− 1.57 ± 0.17
	H^η21/η22^	6.892	− 0.026 ± 0.006	− 0.14 ± 0.04	5.38 ± 1.98
Asn	N^δ^	113.25	7.7 ± 0.2	− 3.2 ± 0.9	− 0.42 ± 0.12
	H^δ21 b^	7.691	0.14 ± 0.01	0.25 ± 0.05	1.79 ± 0.38
	H^δ22 b^	6.990	0.39 ± 0.01	− 0.07 ± 0.05	− 0.18 ± 0.13
Gln	N^ε^	112.93	7.5 ± 0.1	− 5.7 ± 0.7	− 0.76 ± 0.09
	H^ε21 b^	7.639	0.09 ± 0.01	0.16 ± 0.05	1.78 ± 0.59
	H^ε22 b^	6.939	0.41 ± 0.01	− 0.28 ± 0.05	− 0.68 ± 0.12
His	N^δ1^	175.84	0.216 ± 0.005	− 0.19 ± 0.02	− 0.88 ± 0.09
(pH 4.0)	N^ε2^	173.14	2.08 ± 0.06	− 4.7 ± 0.3	− 2.26 ± 0.16
Lys	N^ζ^	32.61	3.0 ± 0.3	− 2.0 ± 1.5	− 0.67 ± 0.51
	(H^ζ^)_3_^+^	7.57	− 0.44 ± 0.03	0.2 ± 0.2	− 0.45 ± 0.46
Trp	N^ε^	129.51	2.84 ± 0.08	− 1.4 ± 0.3	− 0.49 ± 0.11
	H^ε1^	10.197	− 0.24 ± 0.01	0.63 ± 0.05	− 2.63 ± 0.24
Mean^c^	N	107.1 (47.8)	4.0 (2.6)	− 4.0 (2.7)	− 1.15 (0.71)
	H	7.52 (1.08)	0.023 (0.277)	− 0.067 (0.275)	− 0.57 (2.28)

^a^*δ*^0^, *B*_1_, and *B*_2_ were obtained by a fit of the data to Eq. 1. The errors correspond to a confidence level of 95%. Experimental conditions see Table 2. A *B*_2_/*B*_1_-value is given in brackets when its error is equal/larger than the value itself

^b^Stereospecific assignments from Harsch et al. (2013)

^c^Mean values with standard deviation in brackets

#### Imidazole nitrogen atoms of histidine

The histidine N^*δ*1^ and N^ε2^ resonances were assigned by [^1^H,^15^N]-HSQC spectroscopy using the two-bond coupling to the ring protons. In agreement with this assignment are the chemical shift values given by Platzer et al. (2014) for Ac-GHG-NH_2_. In addition, a stronger pH dependence of chemical shifts is expected and observed for N^*δ*1^.

### Pressure dependence of ^1^H chemical shifts of side chain protons bound to a carbon atom

The resonances of side chain protons bound to a carbon atom are listed in Tables 2 and 3. Here, besides long range structural effects the pressure response is expected to depend on the type of the group the proton is attached to as well as on the position in the side chain.

#### Pressure dependent shifts of H^β^-protons

**Fig. 1:**
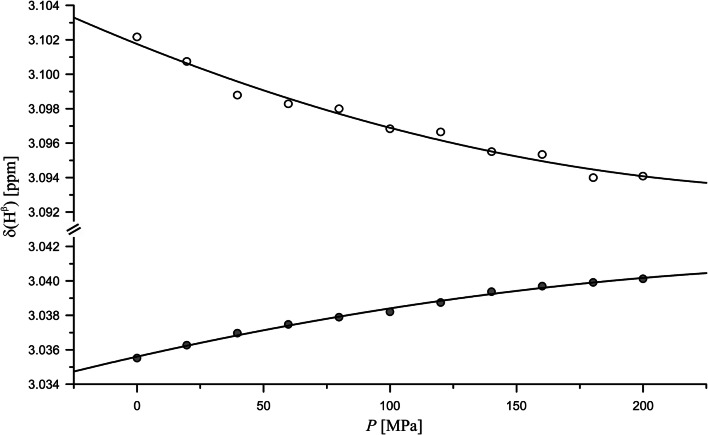
Pressure dependence of chemical shifts of the histidine H^β^- resonances in Ac-GGHA-NH_2_. Experimental conditions and fit parameters see Table 2, pH 8.5, temperature 283 K. (filled circle) H^β2^, (open circle) H^β3^ according to the tentative stereospecific assignment obtained by comparison with the SAIL amino acids

As an example, Fig. 1 shows the pressure response of the H^β^-resonances of histidine in the tetrapeptide that is clearly non-linear. Such a deviation from the linearity is observed for most of the other H^β^-resonances. The size and direction of the pressure induced shifts is strongly dependent on the amino acid under consideration. In Table 2 the parameters *δ*_0_, *B*_1_ and *B*_2_ obtained from a fit of the data are summarized. Most of the resonances show an upfield shift with pressure. In addition, the sign of the second order pressure coefficient *B*_2_ is opposite to that of the first order coefficient *B*_1_ for all values with a reasonable error estimate (Table 2). This leads to a saturation-like behavior where the pressure response gets weaker with higher pressures. In some residues, the two methylene resonances move in different directions with pressure, namely in the aromatic residues His, Phe, and Tyr as well as in Glu and cysteine (see e.g. Fig. 1). According to the SAIL data, the H^β2^-resonances are always shifted upfield relative to the H^β3^-resonances and have a negative first order coefficient *B*_1_. In contrast, the H^β3^-resonances have a *B*_1_ > 0, experiencing a downfield shift in the low pressure range. As a result, the two resonances become further separated with pressure. Since the absolute value of the first order pressure coefficient of the H^β2^-resonances is always larger than that of the H^β3^-resonances, the center of gravity of the two resonances moves upfield with pressure as also observed for all other unresolved methylene resonances except Asn and Asp. Here, both methylene resonances move downfield with pressure. This is probably due to the close-by carbonyl group of the side chain. In contrast to most of the methylene resonances, the resonances of the methine groups of Val and Thr shift downfield with pressure.

#### Pressure dependent shifts of side chain H^γ^-, H^*δ*^-, and H^ε^-methylene protons

**Fig. 2:**
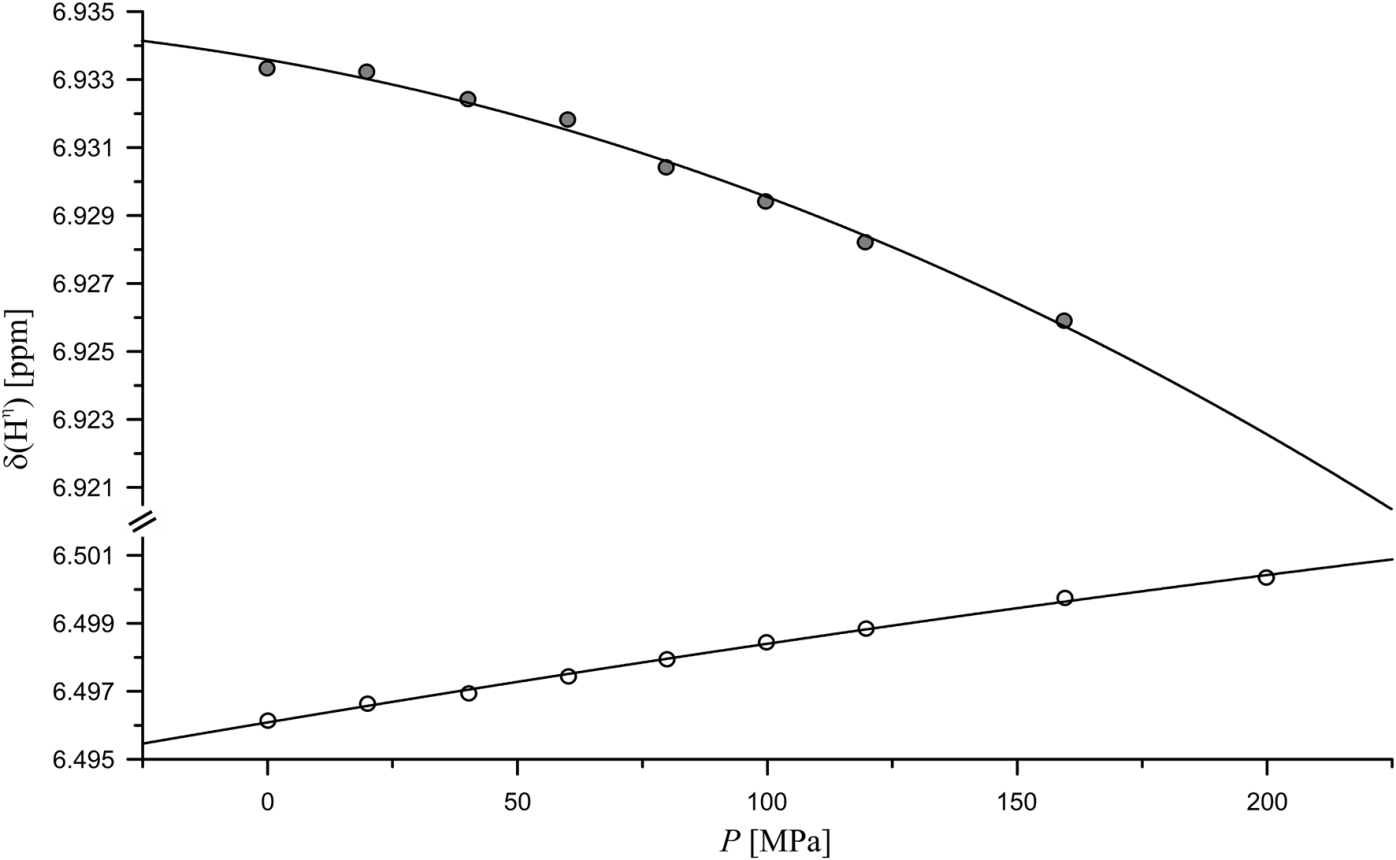
Pressure dependence of the guanidino resonances in Ac-GGRA-NH_2_. Experimental conditions and fit parameters see Table 4, pH 6.7, temperature 283 K, (open circle) H^η11/η12^, (filled circle) H^η21/η22^

**Fig. 3:**
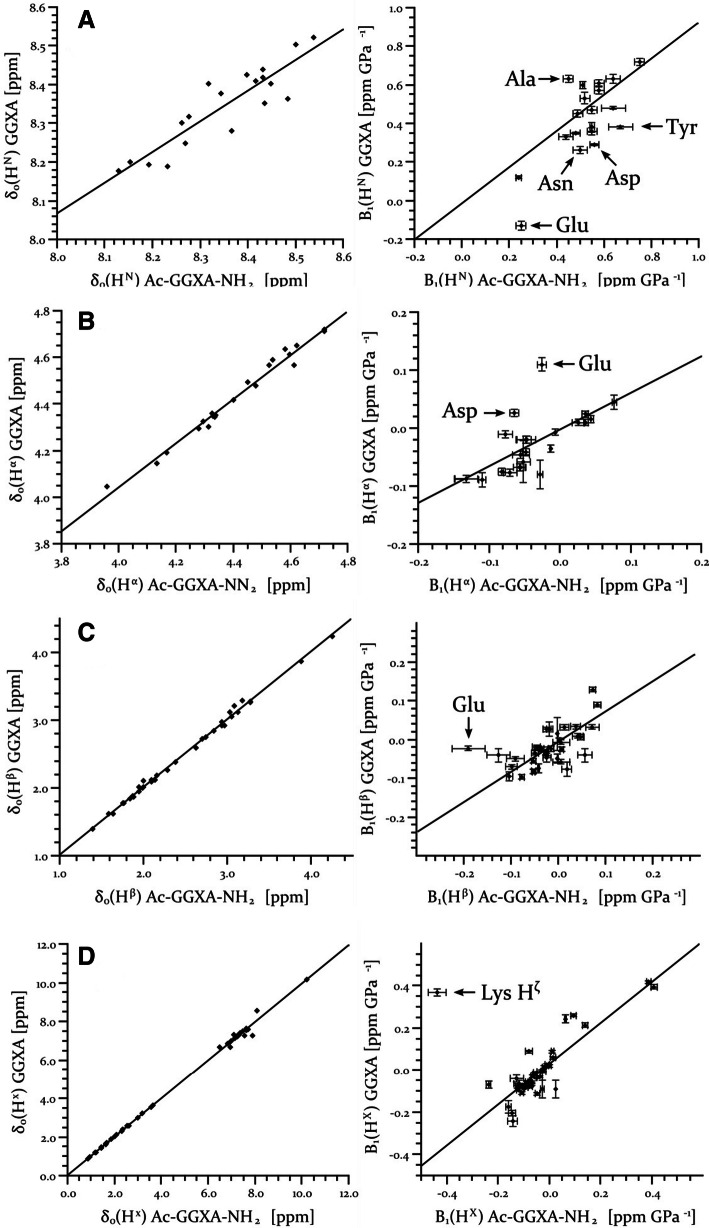
Influence of N- and C- terminal protection on chemical shifts and first order pressure coefficients in the model peptides. The chemical shifts *δ*_0_ and the first order pressure coefficients *B*_1_ of side chain protons of GGXA (Arnold et al.. 2002) at pH 5.4 und T = 305 K are plotted versus those of Ac-GGXA-NH_2_ at pH 6.7 and 283 K. **a** H^N^, **b** H^α^, **c** H^β^, **d** H^X^ with X other side chain protons. The linear correlation coefficients for *δ*_0_ for groups A to D are 0.90, 0.99, 0.99, and 0.99, respectively. For *B*_1_ they are 0.74, 0.65

The proton resonances of all γ-, δ-, and ε-methylene groups have a negative first order pressure coefficient (*B*_*1*_ < 0) and therefore shift upfield with pressure (Table 3). Most of them show a saturation-like behavior (*B*_*2*_/*B*_*1*_ < 0). Omitting all resonances with an uncertainty equal/larger the value itself (values in brackets in Figs. 2 and 3), significant exceptions are the H^γ2^- and H^γ3^-resonances of Glu and the H^γ13^-resonance of Ile.

#### Pressure response of methine protons in γ-position and methyl protons

The methine resonances of Leu and Thr shift again upfield with pressure (*B*_*1*_ < 0) as already described for the methine group of valine. Thr shows a clear saturation-like behavior since *B*_*2*_ is positive. For Leu the error of is so large that a positive or negative sign of *B*_*1*_ is allowed within the limits of error. The methyl resonances of Ile, Leu, Val, Met, and Thr, shift upfield with pressure and all of them exhibit a saturation like behavior (Table 3). An analogous pressure response is also observed for the methyl group of Ala in β-position (Table 2).

#### Pressure dependent shifts of protons in aromatic ring systems

All ring protons of Phe and Tyr show an upfield shift with pressure (*B*_*1*_ < 0) with a saturation like behavior (*B*_*2*_ > 0) (Table 3). This is also true for most of the ring proton resonances of Trp with exception of the H^*δ*1^ and the H^ζ2^ resonances. The latter resonances have positive first and second order pressure coefficients and shift therefore downfield with pressure. Both resonances are relatively close to the ring nitrogen and may be influenced by the pressure dependent polarization of the NH group. In line with this hypothesis, also the proton resonances of His show an analogous pressure response at pH 4.0 and pH 8.5 with positive first order pressure coefficients. The *B*_2_/*B*_1_-ratio of the H^ε1^ –resonance of His at pH 4.0 is negative but positive at pH 8.5.

### Pressure dependence of chemical shifts of side chain nitrogen and their directly bound hydrogen atoms

The resonances of side chain nitrogen atoms and their directly bonded hydrogen atoms are listed in Table 4. All side chain nitrogen resonances shift downfield with increasing pressure and show a slower increase of chemical shifts at higher pressures since *B*_2_ is always negative. The same behavior was observed earlier (Koehler et al, 2012) for the main chain nitrogen resonances.

#### Pressure response of the arginine guanidino group

The ^1^H as well as the ^15^N resonances of the arginine guanidino group are separated at 283 K and 800 MHz proton frequency and could be assigned stereospecifically (see above). At ambient pressure the H^η1^ and H^η2^ resonances are separated only by 0.44 ppm, the N^η1^ and N^η2^ resonances by 2.12 ppm (Table 4). At increasing pressure, the H^η1^ resonances, that are highfield shifted relative to the H^η2^ resonances, shift downfield and the H^η2^ resonances shift upfield with pressure (Fig. 2). This means that these resonances become less separated at higher pressures. However, compared to their initial chemical shift difference this effect is rather small. Both N^η1^ and N^η2^ resonances first shift downfield with pressure (*B*_1_ > 0). At higher pressures they again become closer because of the more negative second order pressure coefficient of the downfield shifted resonance (Table 4). With higher pressure the corresponding proton resonances get broader probably because of the increased exchange rate with the water. Compared to the N^η^ resonances, the N^ε^ resonance shows with 1.8 ppm GPa^−1^ a several times weaker pressure response that get smaller at high pressure. This is because the second order pressure coefficient has the opposite sign of the first order pressure coefficient. The corresponding proton resonance shifts upfield with pressure; the shift changes get stronger with higher pressures since here both pressure coefficients have the same negative sign.

#### Pressure response of resonances of the nitrogen and its attached protons of the histidine and tryptophan rings

The pressure dependence of the histidine nitrogen resonances could only followed at pH 4.0 with sufficient spectral quality. Under these conditions, the ring is positively charged and the two nitrogen atoms are protonated. The proton as well as the nitrogen resonances have positive *B*_1_-values that is, they shift downfield with pressure in our pressure range. Since the *B*_2_-values have signs opposite to the *B*_1_-values, at higher pressure the downfield shifts are attenuated. The *B*_1_- and *B*_2_-value of the N^ε2^ atom are approximately one order of magnitude larger than those of the N^*δ*1^ that shows only a very small pressure dependence. At higher pH values, histidine exists in two tautomers, the τ- and the π-tautomer, with either the N^ε2^ or the N^*δ*1^ being protonated. At pH 8.5 preferentially the N^ε2^ atom remains protonated and is therefore characterized by relatively small chemical shift changes with pH. In contrast, the N^*δ*1^ atom shows much stronger pH-dependence of its chemical shift (Blomberg et al. 1977; Platzer et al. 2014) because of its deprotonation with increasing pH. This means that the relatively strong chemical shift response of the N^ε2^ atom is not due to a partial deprotonation because of a change of the p*K*_a_ of the histidine ring, as this would primarily influence the N^*δ*1^ shifts. The pressure response of the resonances of the protons directly bound to the ring nitrogens of histidine could not be estimated here since they are exchange broadened considerably.

The nitrogen resonance of Trp shows a chemical shift response similar to that of the histidine N^ε2^. It is characterized by a downfield shift with pressure that is slowed down at very high pressures. With a first order pressure coefficient of − 0.24 ppm GPa^−1^, an upfield shift with pressure of the attached proton is observed that is opposite in direction and smaller than the backbone amide shifts with an average first order pressure coefficient of 0.52 ppm GPa^−1^ (Koehler et al. 2012).

#### Pressure response of the lysyl side chain amino group

The nitrogen resonance of the lysyl amino group is shifting downfield with pressure as all side chain and backbone nitrogen resonances. The corresponding protons shift upfield with pressure, in contrast to the expectation that the a decrease of the N–H bond length with pressure would induce a downfield shift (Wagner et al. 1983; Asakawa et al. 1998; Li et al. 1998). The ^1^H linewidth of the amino group in the non-decoupled spectrum is approximately 62 Hz and is not influenced significantly by pressure at 283 K.

#### Pressure response of the amide group of Asn and Gln

The amide side chain nitrogen resonances of Asn and Gln show the largest pressure response of all side chain nitrogen atoms, with *B*_1_ values of 7.7 and 7.5 ppm GPa^−1^, respectively (Table 4). The observed downfield shifts with pressure are also significantly larger than the average backbone amide nitrogen shifts. Their mean *B*_1_ value is 2.91 ppm; the largest first order coefficient is found for Gly with 3.79 ppm GPa^−1^, still smaller than the side chain amide shifts (Koehler et al. 2012). The amide side chain protons of Asn and Gln were assigned stereospecifically earlier (Harsch et al. 2013). Both proton resonances shift downfield with pressure. The two protons show a different pressure response with a larger shift for H^δ22^ in Asn as well as for H^ε22^ in Gln. In HPr from *S. carnosus* almost all (7 out of 9) Asn and Gln amide protons could be assigned stereospecifically by 3D-NOESY spectroscopy and their pressure response could be analysed (Kalbitzer et al. 2000). In all side chains the first order pressure coefficients of the two protons are positive as in the tetrapeptide. At 298 K, except of Asn38 the *B*_1_-values of the upfield shifted resonance (H^δ22^ in Asn and H^ε22^ in Gln) are much larger than the downfield shifted resonances. However, at 278 K also in Asn38 the *B*_1_-value of H^δ22^ is larger than that of H^δ21^ indicating a temperature induced exchange averaging of the two values. Also the nitrogen first order pressure coefficients are relatively large and positive (average 7.67 ppm GPa^−1^ at 278 K). This value is very close to 7.6 ppm GPa^−1^ at 283 K, the mean value for Asn and Gln in our random-coil model although HPr is a quite rigid, well-folded protein. The hydrogen bonding expected in the protein appears to have no larger effect on the amide nitrogen pressure response. This is different for the hydrogen resonances: Here, the mean values for HPr at 278 K are with 0.22 and 1.11 ppm GPa^−1^ significantly larger than 0.12 and 0.40 ppm GPa^−1^ in the tetra peptide at 283 K indicating pressure induced changes in hydrogen bond lengths. The pressure coefficients of the amide side chains in the tetrapeptides predict that at lower pressures the shift difference between the two resonances decreases but increases again at pressures higher than 350 to 400 MPa, since the second order coefficients have different signs. It is expected that a similar behavior is found in the protein but no second order pressure coefficients have been determined here.

### Influence of the N- and C-terminal protection on the pressure response

Protection of the N- and C-terminus of tetrapeptides by acetylation and amidation may influence also the pressure response of amino acid Xxx in position 3. The chemical shifts at ambient pressure *δ*_0_ and the first order pressure coefficients *B*_1_ of different groups of atoms of the unprotected tetrapeptides are plotted against the corresponding values in the protected tetrapeptides in Fig. 3. For the individual chemical shifts *δ*_0_ of the main chain amide protons significant deviations from the correlation line are observed, although the Pearson correlation coefficient is with 0.90 still quite high. For the other three groups of atoms investigated here (H^α^, H^β^, H^X^) (Fig. 3) almost perfect correlations of the chemical shifts at ambient pressure are observed with correlation coefficients of 0.99 indicating that they are not influenced significantly by the protecting groups. The variations of the H^N^-shifts may mainly be due to the different experimental temperatures in the two data sets, since the temperature dependence of these shifts is known to vary from amino acid to amino acid (Jimenez et al. 1986; Kjaergaard et al.2011a). In general, for the first order pressure coefficients much larger deviations from a linear correlation are observed. A simple reason for these deviations may be that only the pressure response of the H^N^-resonances was fitted by a second degree polynomial by Arnold et al. (2002) but that of the other proton resonances only by a first degree polynomial. In contrast, in the present paper the data quality was high enough to fit all data with a second degree polynomial. In addition, temperature and pH is different in the two studies and protected tetrapeptides are compared with unprotected tetrapeptides where the termini are at least partly charged. In agreement with the effect of the fitting procedure, the correlation of the *B*_1_-values of the amide proton resonances is with 0.74 significantly higher than 0.65, 0.61, and 0.57 found for the H^α^-, H^β^-, and the H^X^- (X, other side chain protons than H^α^ or H^β^) resonances, respectively (Fig. 3). A few amino acids show deviations of the first order pressure coefficients from the correlation line, the largest deviations are observed for the H^α^ of Asp and Glu, the H^β^ and the H^ζ^ of Lys (Fig. 3). For Asp and Glu pH-dependent differences in protonation states of the carboxyl groups may be the reason and/or the interaction of the charged side chain with the charged N-or C-termini in the unprotected tetrapeptides. For H^ζ^ of Lys only the latter effect can apply. For GGEA the effect of the terminal charges on the pressure response was already published by Kremer et al. (2003), where the modification of the C-terminal carboxyl group strongly changes the pressure response. However, also polar uncharged residues such as Asn or Tyr show a strong effect on their pressure response when the terminal groups are modified. This again stresses the effects of the charged groups of the termini on the pressure response.

### Neighborhood effects on the pressure response

The chemical shifts of a given amino acid in random-coil peptides are dependent on the next neighbors in the sequence. They can be corrected by using a simple additive model (Braun et al. 1994; Wishart et al. 1995a; Schwarzinger et al. 2001; Tamiola et al. 2010; Kjaergaard et al. 2011a; Kjaergaard and Poulsen 2011b). A similar effect from neighboring amino acids in the sequence can be expected for the pressure response. The data were evaluated analogously to the method applied by Schwarzinger et al. (2001) to the pentapeptide Ac-GGXGG-NH_2_ in 8 M urea at pH 2.3 and 293 K. However, since we have a tetrapeptide, only the correction factors ${C}_{\mathrm{1,2}}^{-1,a}$(*y*), ${C}_{\mathrm{1,2}}^{+1,a}$(*y*), and ${C}_{\mathrm{1,2}}^{+2,a}$(*y*) for atoms *a* could be determined (see “Materials and methods” Section) with ${C}_{\mathrm{1,2}}^{-j,a}$(*y*) correction factors for atom *a* in amino acid X in position *i* when amino acid *y* is located at position *i* + *j* in the sequence. The mean sequence correction factors calculated in our peptide for H^N^ and H^α^ for the shifts at ambient pressure agree well with those obtained by the others groups. They are − 0.03, − 0.08, and 0.09 ppm for H^N^ and − 0.03, − 0.03, − 0.03 ppm for H^α^ for Gly1, Gly2, and Ala4, respectively. In Ac-GGXGG-NH_2_, they are − 0.01, − 0.05, 0.15 ppm for H^N^ and − 0.03 − 0.02, − 0.03 ppm for H^α^ for Gly1, Gly2, and Gly4 as reported by Schwarzinger et al. (2001). The average correction factors reported by Kjaergaard et al. (2011a) are even closer to our values with − 0.02, − 0.07, 0.09 ppm for H^N^ and − 0.02, − -0.02, − 0.03 ppm for H^α^ for Gly1, Gly2, and Gly4. However, the individual values correlate relatively weakly between all three data sets. This is probably due to different experimental conditions, especially the urea concentration, the pH, and the experimental temperatures. Most of the correction factors for the first order pressure coefficients of amide backbone protons are positive (Table 5). The strongest effects on the pressure response are observed when the amino acid directly preceding amino acid X is not a Gly. In average, *B*_1_ is increased by 0.08 ppm GPa^−1^ and *B*_2_ decreased by 0.08 ppm GPa^−2^. Since also all experimental first order coefficients of amide protons are positive (Koehler et al. 2012), the positive correction factor leads to a larger downfield shift with pressure except of Ser, His and Gln that cause a quite small decrease of the pressure response. The largest corrections are observed for aromatic residues and amino acids with long side chains in position i−1. The maximum correction for *B*_1_ is required for Leu with 0.18 ppm GPa^−1^. The effect of amino acids following amino acid X is in the average smaller than that due to the preceding amino acid. It is similar in position i + 1 and i + 2. For the amino acid in position i + 1 the average corrections for the first order and second order pressure coefficient are 0.01 GPa^−1^ and − 0.05 ppm GPa^−2^; in position i + 2 they are 0.04 GPa^−1^ and − 0.05 ppm GPa^−2^.

**Table 5:** Sequence dependent correction factors for the pressure coefficients *B*_1_ and *B*_2_

Yyy	Atom	*B*_1_			*B*_2_		
		(ppm GPa^−1^)			(ppm GPa^−2^)		
		${C}_{1}^{-1,a}$	${C}_{1}^{+1,a}$	${C}_{1}^{+2,a}$	${C}_{2}^{-1,a}$	${C}_{2}^{+1,a}$	${C}_{2}^{+2,a}$
Ala	H^N^	0.07	0.08	0.02	− 0.11	− 0.19	0.02
	H^α^	− 0.02	− 0.01	0.01	0.05	− 0.02	0.01
	H^β^_3_	0.00			− 0.01		
Arg	H^N^	0.01	0.04	0.00	0.12	0.04	0.01
	H^α^	− 0.03	− 0.05	− 0.01	0.02	0.10	0.05
	H^β^_3_	− 0.01			0.02		
Asn	H^N^	0.05	0.01	− 0.01	− 0.02	− 0.09	0.02
	H^α^	0.01	− 0.02	− 0.02	− 0.08	0.01	0.12
	H^β^_3_	− 0.02			0.03		
Asp	H^N^	0.06	0.21	0.03	− 0.19	0.34	0.01
	H^α^	0.01	− 0.05	− 0.03	0.00	0.12	0.06
	H^β^_3_	− 0.01			0.01		
Cys	H^N^	0.02	− 0.08	0.11	− 0.12	0.00	− 0.02
	H^α^	0.01	− 0.01	− 0.01	− 0.09	− 0.01	− 0.01
	H^β^_3_	0.00			− 0.01		
Gln	H^N^	− 0.04	− 0.02	0.01	0.32	0.17	− 0.02
	H^α^	− 0.05	− 0.02	0.00	0.08	− 0.02	0.01
	H^β^_3_	0.00			− 0.01		
Glu	H^N^	0.11	0.04	0.15	− 0.27	− 0.08	− 0.15
	H^α^	− 0.03	− 0.02	− 0.01	0.02	− 0.01	0.01
	H^β^_3_	− 0.01			0.00		
Gly	H^N^	0	0	0	0	0	0
	H^α^	0	0	0	0	0	0
	H^β^_3_	0			0		
His	H^N^	0.00	0.04	0.01	− 0.12	− 0.08	− 0.03
(pH 4.0)	H^α^	− 0.03	0.00	− 0.01	0.03	− 0.04	0.00
	H^β^_3_	− 0.02				0.01	
Ile	H^N^	0.14	− 0.04	0.05	− 0.07	− 0.09	− 0.36
	H^α^	− 0.04	− 0.04	0.00	0.01	0.13	0.02
	H^β^_3_	0.01			− 0.03		
Leu	H^N^	0.18	0.10	0.03	− 0.13	− 0.15	− 0.05
	H^α^	− 0.03	− 0.02	0.01	0.02	0.00	− 0.02
	H^β^_3_	− 0.01			0.02		
Lys	H^N^	0.12	0.09	0.05	− 0.03	− 0.36	− 0.18
	H^α^	− 0.02	0.02	0.01	0.07	− 0.07	0.00
	H^β^_3_	0.00			− 0.01		
Met	H^N^	0.14	0.07	0.01	− 0.17	− 0.13	0.01
	H^α^	− 0.03	0.00	0.00	0.06	0.03	0.01
	H^β^_3_	0.00			− 0.01		
Phe	H^N^	0.10	− 0.09	0.02	0.01	− 0.25	0.07
	H^α^	− 0.04	− 0.02	− 0.02	0.14	− 0.01	− 0.01
	H^β^_3_	− 0.01			0.03		
Pro_trans_	H^N^	0.13	− 0.06	0.25	− 0.21	− 0.06	− 0.41
	H^α^	− 0.03	− 0.01	0.00	0.06	− 0.01	− 0.01
	H^β^_3_	0.01			− 0.02		
Ser	H^N^	− 0.02	0.02	0.04	− 0.03	− 0.11	− 0.22
	H^α^	− 0.01	0.00	0.00	0.03	0.00	− 0.01
	H^β^_3_	− 0.01			− 0.01		
Thr	H^N^	0.09	− 0.02	0.00	− 0.24	0.11	− 0.03
	H^α^	− 0.03	− 0.01	0.00	0.02	− 0.03	0.00
	H^β^_3_	0.00			0.00		
Trp	H^N^	0.16	0.03	0.09	− 0.25	− 0.07	− 0.08
	H^α^	− 0.14	− 0.03	0.04	0.06	− 0.03	− 0.09
	H^β^_3_	− 0.12			− 0.05		
Tyr	H^N^	0.16	− 0.13	− 0.02	− 0.24	− 0.21	0.26
	H^α^	− 0.04	− 0.03	− 0.01	0.07	0.01	0.04
	H^β^_3_	− 0.01			0.03		
Val	H^N^	0.08	− 0.13	− 0.02	0.05	0.21	0.13
	H^α^	− 0.05	− 0.01	− 0.01	0.05	− 0.03	0.04
	H^β^_3_	0.01			0.00		
Mean	H^N^	0.08	0.01	0.04	− 0.08	− 0.05	− 0.05
	H^α^	− 0.03	− 0.02	0.00	0.03	0.01	0.01
	H^β^_3_	− 0.01			0.01		

Sequence dependent correction factors ${C}_{1}^{-1,a}$ for the pressure coefficients *B*_1_ and *B*_2_ of atom *a* in amino acid Xxx in position *i* by amino acid Yyy in position *i* + *j*. For their definitions, see “Materials and methods” Section

The corrections for the H^α^ pressure induced shifts are substantially smaller than those for the amide protons. Again, the effects of the preceding amino acid are stronger than the effects of the succeeding amino acids. The average corrections required for *B*_1_ and *B*_2_ by amino acids in position i−1 are − 0.03 GPa^−1^ and 0.03 ppm GPa^−2^, respectively. The corresponding values for the succeeding amino acids are − 0.02 GPa^−1^ and 0.01 ppm GPa^−2^ (i + 1), and 0.00 GPa^−1^ and 0.01 ppm GPa^−2^ (i + 2).

Since most of the H^α^ resonances shift upfield with pressure (Beck Erlach et al. 2016), the negative correction factors for *B*_1_ values intensify the downfield shift. It has been observed earlier and also verified here for the side chain atoms that most second order coefficients have an opposite sign relative to the first order coefficient (Beck Erlach et al. 2016). The same is true for the correction factors, meaning that in most cases the curvature is enhanced by amino acids other than Gly in the neighborhood.

From our data set, also the correction factors ${C}_{\mathrm{1,2}}^{-1,a}$(*y*) for the β-methyl group of Ala can be derived. The correction factors ${C}_{1}^{-1,a}$ vary between − 0.12 and 0.01 ppm GPa^−1^ and the correction factors ${C}_{2}^{-1,a}$ between − 0.05 and 0.03 ppm GPa^−2^ and thus are of the same order of magnitude than the corresponding *B*_1_ and *B*_2_ values themselves of − 0.022 ppm GPa^−1^ and 0.02 ppm GPa^−2^, respectively (Table 3 and 4).

### Correlation between the second and first order pressure coefficients for different groups of side chain atoms

**Fig. 4:**
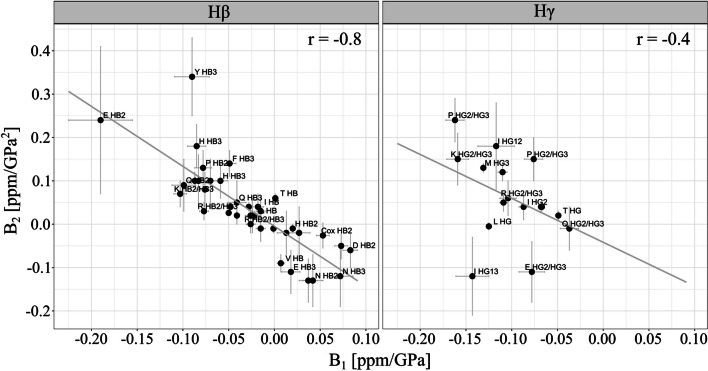
Correlations between the first and second order pressure coefficients *B*_1_ and *B*_2_ of β- and ^γ^-proton resonances. The Pearson correlation coefficients r of the H^β^- and H^γ^-resonances are -0.8 and -0.4, the corresponding slopes − 1.38 ± 0.159 and − 1.01 ± 0.645 GPa^−1^, respectively

As shown earlier (Beck Erlach et al. 2014), under certain conditions the ratio of *B*_2_/*B*_1_ is related to the local compressibility. When the pressure response can be described by a two-state model with a free energy difference |Δ*G/*2*RT*|< < 1, *B*_2_/*B*_1_ equals—½ Δβ*′*/Δ*V*. Note that in the cited paper the definition of the second order pressure coefficient was different leading to a factor of two relative to *B*_2_ defined by Eq. 1 in the present paper. Δβ*’* is the difference of the partial molar compressibility factors and Δ*V* the difference in the partial molar volumes. If this process describes the whole tetrapeptide, the obtained values should be identical for all atoms of a given tetrapeptide, if it describes a general feature of all tetrapeptides, e. g. the properties of the surrounding water shell, it should have the same value for all atoms and tetrapeptides within the limits of error. Figure 4 shows a plot *B*_2_ as a function of *B*_1_ for the H^β^- and the H^γ^-resonances. Whereas the two quantities are relatively well correlated for H^β^-resonances with a correlation coefficient of − 0.82, the correlations for the H^γ^-resonances and the H^*δ*^-resonances are much smaller with correlation coefficients of − 0.48 and − 0.21, respectively, probably, since they represent a chemically more inhomogeneous group of atoms (Table 6). For the other side chain proton resonances the correlation is again larger, probably since these resonances form again a more homogenous group.

**Table 6:** Position specific correlation analysis of main and side chain resonances

Atom	# Atoms	*B*_2_*/B*_1_	Correlation
		(GPa^−1^)	Coefficient
C’	23	− 1.07 ± 0.22	− 0.73
C^α^	23	− 1.25 ± 0.38	− 0.59
C^β^	22	− 0.55 ± 0.08	− 0.84
C^γ^	20	− 0.64 ± 0.09	− 0.85
C^*δ*^	17	− 0.90 ± 0.16	− 0.82
C^ε^	10	− 0.73 ± 0.15	− 0.86
C^ζ^	5	− 0.54 ± 0.07	− 0.98
H^N^	19	− 1.45 ± 0.25	− 0.82
H^α^	24	− 1.13 ± 0.23	− 0.73
H^β^	40	− 1.39 ± 0.16	− 0.82
H^γ^	21	− 1.22 ± 0.51	− 0.48
H^*δ*^	18	− 0.18 ± 0.21	− 0.21
H^ε^	12	− 0.79 ± 0.32	− 0.61
H^ζ^	4	− 0.29 ± 0.04	− 0.99
N	22	− 1.13 ± 0.28	− 0.67
N^ε^	4	− 0.35 ± 0.42	− 0.51

The correlation coefficients between *B*_1_ and *B*_2_ and the average *B*_2_ to *B*_1_ ratio of the side chain proton and nitrogen resonances were calculated from the data presented in this paper (Tables 2, 3 and 4), the side chain carbon data (Beck Erlach et al. 2017) and the main chain data (Koehler et al. 2012; Beck Erlach et al. 2016) were reevaluated for this paper. The data from the cis/trans-isomers of proline and from cysteine and cystine were used separately, leading to higher numbers of atoms than expected for 20 amino acids

We have reevaluated the data partly presented by Beck Erlach et al. (2016, 2017) and Koehler et al. (2012). Relative high negative correlation ≤ − 0.82 are found for the side chain carbons (Table 6). Also quite high negative correlations were found for the main chain atoms H^N^, H^α^, C^α^, N, and C′ (Table 6, Beck Erlach et al. 2016) that form again chemically more homogeneous groups similar to the H^β^-resonances as already discussed. Ordering the resonances according to their chemical groups, e. g. methylene and methyl groups leads to similar correlation coefficients for *B*_1_ and *B*_2_ as found for ordering them according to their position in the side chain, indicating that assignment to a given chemical group represents a property independent of the position.

The slopes of the plot of *B*_2_ as function of *B*_1_ (see e. g. Fig. 4) corresponds to their ratios for different positions in the amino acids and different groups. They are listed in Tables 6 and 7.

**Table 7:** Group specific correlation analysis of side chain resonances

Atom	# Atoms	*B*_2_/*B*_1_	Correlation
		(GPa^−1^)	Coefficient
C	5	− 0.88 ± 0.11	− 0.98
C (Ring)	7	− 1.11 ± 1.15	− 0.40
CH (ring)	19	− 1.46 ± 0.21	− 0.86
CH_2_	32	− 0.49 ± 0.08	− 0.73
CH_3_	9	− 1.28 ± 0.78	− 0.53
HC (ring)	14	− 0.84 ± 0.38	− 0.54
HC	4	− 0.04 ± 0.76	− 0.04
H_2_C	62	− 1.02 ± 0.15	− 0.65
H_3_C	9	− 0.59 ± 0.20	− 0.74
H_2_N	6	− 0.42 ± 0.48	− 0.40
NH_2_	4	1.39 ± 0.91	0.74

For details see Table 6

For the side chain protons they vary in the range of − 0.18 (H^*δ*^) and − 1.39 GPa^−1^ (H^β^), for the side chain carbons in the range of − 0.45 (C^ζ^) and − 0.90 GPa^−1^ (C^*δ*^), for the main chain atoms between -1.07 (C′) and − 1.45 GPa^−1^ (H^N^) (Table 6). The values obtained from the slope are much more reliable than just the means calculated from the individual values in Tables 2 and 3 because the errors of the individual values are sometimes quite large. As an example, one would obtain mean values of *B*_2_/*B*_1_ of 0.4 ± 2 and − 0.31 ± 1.12 GPa^−1^ for the H^β^- and the H^γ^-resonances (Tables 2 and 3), respectively, very different to the values given in Table 6. When considering distinct chemical groups, similar *B*_2_/*B*_1_-ratios are obtained. A significant exception is the pressure response of the nitrogen resonances of side chain amide groups where the *B*_2_/*B*_1_-ratio is positive with 1.39 GPa^−1^. Here, a specific pressure effect may become visible, e. g. the interaction of the NH_2_-group with its C=O-group.

Many *B*_2_/*B*_1_-ratios for the different groups and positions are the same within the limits of error indicating that at least partly a global two-site exchange may be involved in the observed pressure response.

### Application of pressure coefficients of random-coil peptides

The simplest application of the random-coil pressure coefficients is the interpretation of pressure dependent chemical shifts of polypeptides. If the experimental shifts of a stretch of the sequence at ambient pressure are close to those predicted from the random-coil parameters, the probability is high that this region is disordered. This is even more likely when its pressure response approximates that of a random-coil model peptide as defined by its pressure coefficients. If this is true for any pressure than the probability is very high. It has been proposed earlier for peptides at ambient pressure, that also a secondary structure propensity can be derived from the chemical shift difference of the actual values from random-coil values (Yao et al. 1997). This procedure can now also be applied at data recorded at high pressure since the random-coil shifts at any pressure are now known. Such a prediction of the secondary structure propensity would allow a more informative interpretation of NMR spectra of intrinsically disordered proteins (see e.g. Roche et al. 2013).

Another important application of the model peptides is the thermodynamic evaluation of structural transitions of proteins (see e.g. Kalbitzer et al. 2013a; Kalbitzer 2015). When a protein exists in N structural states with M ≤ N states in fast exchange and the other states in slow or intermediate exchange, the pressure dependence of the chemical shift of a certain atom *k* (Baskaran et al. 2010) is given by

5 $$\begin{aligned}\langle {\delta }^{k}\rangle &={\sum }_{j=1}^{M}{p}_{j}\left(\Delta P\right){\delta }_{j}^{k}\left(\Delta P\right)=\frac{{\sum }_{j=1}^{M}{\delta }_{j}^{k}\left(\Delta P\right){e}^{\frac{-\Delta {G}_{1j}\left(\Delta P\right)}{RT}}}{{\sum }_{j=1}^{M}{e}^{\frac{-\Delta {G}_{1j}\left(\Delta P\right)}{RT}}}\\&=\frac{{\delta }_{1}^{k}+{\sum }_{j=2}^{M}{\delta }_{j}^{k}\left(\Delta P\right){\prod }_{k=1}^{j-1}{e}^{\frac{-\Delta {G}_{k\left(k+1\right)}\left(\Delta P\right)}{RT}}}{1+{\sum }_{j=2}^{M}{\prod }_{k=1}^{j-1}{e}^{\frac{-\Delta {G}_{k\left(k+1\right)}\left(\Delta P\right)}{RT}}}\end{aligned}$$

with *p*_j_ the probability for state *j*; Δ*P* = *P*-*P*_0_ the difference of the actual pressure and the initial pressure (usually ambient pressure); ${\delta }_{j}^{k}(\Delta P)$ the chemical shift of atom *k* in state *j* as function of Δ*P*; Δ*G*_ij_ (Δ*P*) the difference between the free energy of state *i* and state *j* as function of Δ*P*; *R* the gas constant and *T* the absolute temperature. One has always to be aware that the terms slow, intermediate, and fast are measured relative to the NMR-time scale (essentially the chemical shift difference of the nucleus in different states) that may be different for any observed nucleus although only one global transition with a fixed absolute time scale is involved. In addition, note that the chemical shift ${\delta }_{j}^{k}$ generally is a function of pressure since the compression of the molecule per se causes a change of all chemical shift. The pressure dependence of Δ*G*_ij_ (Heremans and Smeller 1998) is given by

6 $$\Delta {G}_{ij}\left(\Delta P\right)=\Delta {G}_{ij}\left(0\right)+\Delta {V}_{ij}^{0}\Delta P+\frac{1}{2}\frac{\partial {\Delta V}_{ij}^{0}}{\partial P}{\left(\Delta P\right)}^{2}$$

with $\Delta {V}_{ij}^{0}$ the difference in the molar volume of state *i* and state *j*.

The function describing the change of ${\delta }_{j}^{k}$ by increasing pressure is not known and has to be approximated. It is caused by the anisotropic compression of the structure in state *j* combined with an amino acid specific chemical shift response. The functional dependence on pressure is not known a priori but the random coil data suggest that in general the pressure dependence of chemical shifts is non-linear and amino acid and atom specific. As in the case of random-coil peptides at least a second degree Taylor polynomial may be required (Eq. 1). A full fit of the compression effects on the chemical shifts ${\delta }_{j}^{k}\left(P\right)$ would then require three additional parameters per atom and conformational state to be fitted to the data together with three global parameters per transition required for the thermodynamic analysis, that is

7 $$\langle {\delta }^{k}\rangle =\frac{{\sum }_{j=1}^{M}{(\delta }_{0,j}^{k}+{B}_{1,j}^{k}\Delta P+ {B}_{2,j}^{k}{{(\Delta P)}^{2})e}^{\frac{-\Delta {G}_{1j}}{RT}}}{{\sum }_{j=1}^{M}{e}^{\frac{-\Delta {G}_{1j}}{RT}}}$$

It is obvious that the number of parameters is much too large to allow a stable fit of the data. With the assumption that the compression effects are (almost) identical in the different states, Eq. 7 can be simplified with the state independent pressure coefficients ${B}_{1}^{k}$ and ${B}_{2}^{k}$ to

8 $$\begin{aligned}\langle {\delta }^{k}\rangle &=\frac{{\sum }_{j=1}^{M}{\delta }_{0,j}^{k}{e}^{\frac{-\Delta {G}_{1j}}{RT}}}{{\sum }_{j=1}^{M}{e}^{\frac{-\Delta {G}_{1j}}{RT}}}+\frac{{(B}_{1}^{k}\Delta P+ {B}_{2}^{k}{(\Delta P)}^{2}){\sum }_{j=1}^{M}{e}^{\frac{-\Delta {G}_{1j}}{RT}}}{{\sum }_{j=1}^{M}{e}^{\frac{-\Delta {G}_{1j}}{RT}}}\\&=\frac{{\sum }_{j=1}^{M}{\delta }_{0,j}^{k}{e}^{\frac{-\Delta {G}_{1j}}{RT}}}{{\sum }_{j=1}^{M}{e}^{\frac{-\Delta {G}_{1j}}{RT}}}+{(B}_{1}^{k}\Delta P+ {B}_{2}^{k}{(\Delta P)}^{2})\end{aligned}$$

A further simplification can be introduced by assuming that the pressure coefficients can be approximated by the corresponding random-coil values. This is equivalent to subtracting the function ${(B}_{1}^{k}\Delta P+ {B}_{2}^{k}{(\Delta P)}^{2}$ from the experimental chemical shifts before fitting the data as it is often done.

## Conclusions

With this paper, together with the data previously published by Beck Erlach et al. (2016, 2017), we have a complete data set of the pressure response of a random-coil model peptides including all ^1^H-, ^13^C-, and ^15^N -resonances of the main and side chains. For the amide and H^α^-resonances we now also provide a correction for different neighbors in the sequence.

A simple use of this data is to compare the pressure response of model peptides derived here with a protein whose structure is unknown. If the pressure response of the protein or part of the protein is equal to that derived for the random-coil peptides, it can be safely assumed that it is mainly disordered.

The other application is the use of these data for the description of the compression effects observed in a multistate protein as described above. Of course, these random-coil data cannot completely predict these effects since in general the compression effects are also influenced by the three-dimensional structure.

A third application of the high-pressure data is their application for developing theory. At the end only quantum chemical methods will be sufficient to calculate the pressure response of larger peptides. For the peptide bond model N-methyl-acetic acid (NMA) we could show that the pressure dependent chemical shift changes can be predicted rather well for the ^1^H, ^13^C, and ^15^N nuclei (Frach et al. 2016).
